# Impaired foot placement strategy during walking in people with incomplete spinal cord injury

**DOI:** 10.1186/s12984-022-01117-0

**Published:** 2022-12-05

**Authors:** Eline Zwijgers, Edwin H. F. van Asseldonk, Marije Vos-van der Hulst, Alexander C. H. Geurts, Noël L. W. Keijsers

**Affiliations:** 1 Department of Rehabilitation, Donders Institute for Brain, Cognition and Behaviour, Radboud University Medical Center, Nijmegen, The Netherlands; 2 Department of Research, Sint Maartenskliniek, Nijmegen, The Netherlands; 3 Department of Biomechanical Engineering, University of Twente, Enschede, The Netherlands; 4 Department of Rehabilitation, Sint Maartenskliniek, Nijmegen, The Netherlands; 5 Department of Sensorimotor Neuroscience, Donders Institute for Brain, Cognition and Behaviour, Radboud University, Nijmegen, The Netherlands

**Keywords:** Walking, Balance, Spinal cord injury, Foot placement strategy

## Abstract

**Background:**

Impaired balance during walking is a common problem in people with incomplete spinal cord injury (iSCI). To improve walking capacity, it is crucial to characterize balance control and how it is affected in this population. The foot placement strategy, a dominant mechanism to maintain balance in the mediolateral (ML) direction during walking, can be affected in people with iSCI due to impaired sensorimotor control. This study aimed to determine if the ML foot placement strategy is impaired in people with iSCI compared to healthy controls.

**Methods:**

People with iSCI (n = 28) and healthy controls (n = 19) performed a two-minute walk test at a self-paced walking speed on an instrumented treadmill. Healthy controls performed one extra test at a fixed speed set at 50% of their preferred speed. To study the foot placement strategy of a participant, linear regression was used to predict the ML foot placement based on the ML center of mass position and velocity. The accuracy of the foot placement strategy was evaluated by the root mean square error between the predicted and actual foot placements and was referred to as foot placement deviation. Independent t-tests were performed to compare foot placement deviation of people with iSCI versus healthy controls walking at two different walking speeds.

**Results:**

Foot placement deviation was significantly higher in people with iSCI compared to healthy controls independent of walking speed. Participants with iSCI walking in the self-paced condition exhibited 0.40 cm (51%) and 0.33 cm (38%) higher foot placement deviation compared to healthy controls walking in the self-paced and the fixed-speed 50% condition, respectively.

**Conclusions:**

Higher foot placement deviation in people with iSCI indicates an impaired ML foot placement strategy in individuals with iSCI compared to healthy controls.

## Background

Impaired balance during walking is a common problem in people with incomplete spinal cord injury (iSCI) [1]. Indeed, individuals with iSCI experience reduced functional ambulation [2] and increased fall risk [3]. Hence, improving dynamic balance is essential to them. Characterizing balance control during walking and how dynamic balance is affected in people with iSCI is crucial for designing and improving effective intervention strategies. However, only few studies have investigated balance control during walking in people with iSCI [4–9].

Balance control requires coordination of the center of mass (COM) relative to the base of support (BOS). During walking, the relation between the COM and BOS is typically modulated by a combination of the hip, ankle, and foot placement strategy [10, 11]. Basically, the hip and ankle strategies involve adjustments to the COM by rotating the body around the respective joints [10–12]. The foot placement strategy involves adjustments to the BOS by controlling the location and timing of foot placement [10, 11]. The foot placement strategy modulates the relation between the COM and BOS at relatively low actuation costs, because it only requires movement of the swing leg. Consequently, foot placement is the dominant mechanism to maintain balance in the mediolateral (ML) direction during walking in healthy subjects [13, 14].

Literature suggests that ML foot placement is based on COM kinematics [15–19]. This was first observed in simulations, where stable walking was achieved by positioning the foot at a fixed distance lateral to the extrapolated COM (i.e., the COM position adjusted for its velocity) [15]. The relation between ML foot placement and COM kinematics was also observed in experiments investigating foot placement modulation in healthy subjects [16–19]. For example, in the work of Vlutters et al. [18], healthy subjects showed foot placement adjustments proportional to the ML COM velocity when being perturbed in the ML direction during walking. Furthermore, Wang and Srinivasan [19] showed that ML foot placement can be predicted by the ML COM position and velocity, suggesting that the ML foot placement strategy comprises a strong relation between ML COM kinematics and ML foot placement.

Adjusting foot placement based on COM kinematics requires an adequate estimate of the COM state and sufficient ability to move the swing leg. The COM kinematics must be estimated using visual, vestibular, and proprioceptive information [14], which inputs are used to control the placement of the swing leg, for instance, by modulating the activity of the hip abductor muscles to make step adjustments in the ML direction [20]. Because iSCI potentially affects the afference of sensory information as well as the conduct of efferent neural signals to the muscles [1], it may easily impact the ML foot placement strategy. Indeed, impaired foot placement after spinal cord injury has already been suggested by Day and colleagues [4]. Their results revealed higher variability in ML foot placement relative to the COM position in people with iSCI compared to healthy controls. Moreover, in the study of Arora et al. [7], people with iSCI generated less soleus activation in the swing leg after slip perturbations, suggesting impaired muscle control in balance-challenging conditions. Cornwell et al. [6] examined the effect of walking speed on gait stability and concluded that individuals with iSCI were able to maintain lateral stability when walking at a fast speed, even when their lateral balance was challenged. Furthermore, their results suggested a weaker coordination between COM state and lateral foot placement in people with iSCI compared to healthy controls, implying an impaired ML foot placement strategy. However, they instructed participants to maintain their COM within a narrow target lane, which may yield different results than unrestricted walking. Therefore, more research is necessary to evaluate the ML foot placement strategy in people with iSCI during regular straight walking.

The main purpose of this study was to determine if the ML foot placement strategy is impaired in people with iSCI compared to healthy controls. More specifically, this study investigated the relation between ML COM kinematics and ML foot placement during straight walking in both populations. We hypothesized that the ML foot placement strategy would be impaired in people with iSCI [6].

## Methods

### Participants

Participants were people with iSCI that had been referred to the GRAIL (gait real-time analysis interactive lab) training by a rehabilitation physician to improve their gait capacity and dynamic balance. Inclusion criteria were: (1) a motor incomplete spinal cord injury with a traumatic or non-traumatic cause (American spinal injury association impairment scale (AIS) C or D), (2) 6 months post injury, (3) ability to walk in a self-paced mode on the GRAIL without using the handrails, and (4) age ≥ 18 years. Subjects were excluded if they had pre-injury impairments of the nervous system, or lower limbs, or any other impairment that might affect balance control. Healthy controls were included if they were 18 years or older without a history of neurological or musculoskeletal problems. The study was approved by the regional medical ethics committee of Arnhem-Nijmegen (2019–5255). All participants provided written informed consent under the Declaration of Helsinki.

### Data collection

Participants were tested on an instrumented split-belt treadmill (GRAIL, Motek Medical BV, The Netherlands). Kinematic data were acquired using an eight-camera motion capture system (VICON, Oxford, United Kingdom). Reflective markers were placed on 19 anatomical landmarks: 7th cervical vertebra and left and right acromion process, humeral lateral epicondyle, ulnar styloid process, anterior superior iliac spine (ASIS), posterior superior iliac spine (PSIS), femoral lateral epicondyle, lateral malleolus, metatarsal II, and calcaneus. Marker data were sampled at 100 Hz.

### Protocol

All participants performed a two-minute walk test (2MWT) at a self-paced speed on the treadmill. The participants with iSCI performed the 2MWT at the start of their first training session. The speed of the belt was adjusted in real-time to the anterior–posterior position and velocity of the pelvis to allow participants to walk at a self-selected walking speed (self-paced mode), which is a suitable alternative to fixed-speed treadmill walking in gait analysis [21, 22]. In the self-paced mode, walking on the front part of the treadmill results in acceleration proportional to the difference between the pelvis position and middle of the belt, and to the velocity of the pelvis. Likewise, walking on the back part of the treadmill results in deceleration. The participants were instructed to walk at a comfortable walking speed. The healthy controls performed one extra 2MWT at a fixed speed equal to 50% of their mean self-paced walking speed (preferred speed) to analyze the effects of walking speed on their ML foot placement strategy, and because this speed was presumed to be similar to the preferred walking speed of the participants with iSCI [23]. A fixed speed was selected because walking in the self-paced mode at 50% of the preferred speed is challenging, and previous research found no significant differences between self-paced and fixed-speed walking [21, 22]. Before the 2MWTs, participants performed one to four one-minute practice rounds to familiarize themselves with walking on the treadmill. To ensure safety, all participants wore a safety harness attached to a rail on the ceiling, without body weight support.

### Data analysis

Data were processed using MATLAB (R2019b, MathWorks). The first 20 and last 5 seconds of each 2MWT were excluded from the analysis to remove the start and stop phases. Gaps in the ASIS and PSIS marker data were automatically filled using the rigid body method as previously described [24]. Cubic spline fill was used for the remaining markers when a gap was no more than 10 samples. Marker data were filtered with a 4th order zero-phase low-pass Butterworth filter with a cut-off frequency of 20 Hz.

Hip joint centers were estimated using the regression method reported by Dumas et al. [25]. Marker data and hip joint centers were used to estimate the COM location of nine segments (torso and head, upper leg, lower leg and foot, upper arm, forearm and hand) as described by Tisserand et al. [26]. The whole-body COM location was computed using a weighted sum of the segment COM locations. Gaps in the whole-body COM, resulting from gaps in the marker data, were filled using the pattern fill method as described by Camargo et al. [24]. The average location of the ASIS and PSIS markers was used as the donor pattern.

Marker data of the feet were used to detect gait instances [27]. Heel strike was defined as the instant at which the anterior–posterior velocity of the calcaneus marker reversed with respect to the walking direction. Toe-off was defined as the instant at which the velocity of the metatarsal II marker reversed to the positive walking direction. Step width was defined as the distance between the left and right calcaneus marker at the instant of midstance.

To study the foot placement strategy of a participant, linear regression was used to predict the ML foot placement (FP) based on ML COM position and velocity at heel strike [19, 28–30]. We used the following regression equation:

$$FP= {\beta }_{pos}\cdot COM+ {\beta }_{vel}\cdot \dot{COM}+ \varepsilon$$

in which ${\beta }_{pos}$ and ${\beta }_{vel}$ are the regression coefficients of the COM position and velocity, respectively, and $\varepsilon$ the model error. Foot placement was defined as the demeaned ML distance between the left and right calcaneus markers at midstance. The COM position was defined with respect to the calcaneus marker of the stance foot at mid stance, and both predictors were demeaned.

### Outcome measures

The accuracy of the foot placement strategy was evaluated by the root mean square error (RMSE) between the predicted and actual foot placements. The RMSE was selected as primary outcome measure and referred to as foot placement deviation.

To confirm adherence to the foot placement strategy, the goodness of the fit of the linear regression model was evaluated with the coefficient of determination (R^2^), here referred to as foot placement adherence. Substantial adherence to the foot placement strategy was considered when the coefficient of determination was larger than 0.26 [31]. In addition, the within-subject standard deviation (SD) of actual foot placement was determined, because foot placement adherence is influenced by the dispersion of the actual ML foot placement.

Step width was selected as a secondary outcome measure, because wider steps have previously been linked to instability during walking [32, 33] and a reduced foot placement strategy [34].

### Statistical analysis

Participant characteristics of both groups were compared with independent t-tests for continuous variables and Chi-square tests for nominal variables. Foot placement deviation of people with iSCI was compared with values obtained from healthy controls at different walking speeds using independent t-tests, whereas a difference in foot placement deviation between different walking speeds in healthy controls was tested with a dependent t-test. Likewise, group differences in foot placement adherence, in the SD of actual foot placement, and in step width were tested with independent t-tests, whereas differences between different walking speeds within the healthy control group were tested with dependent t-tests. We performed the Student's independent t-test when the assumption of homogeneity of variance was met and the Welch's independent t-test when this assumption was not met (resulting in fractional degrees of freedom). When the assumption of normality was violated, non-parametric equivalent tests were performed. The level of significance (α) was adjusted for the number of tests performed (3) and set at 0.017.

## Results

### Participants

In total, 30 people with iSCI and 19 healthy controls participated. Two persons with iSCI were not included in the analysis due to incomplete marker data, resulting in 28 people with iSCI. Participant characteristics are reported in Table 1. No significant differences in sex and age were found between both groups. The weight and height of the iSCI group were higher compared to controls, but no significant difference in body mass index (BMI) was found between groups (t(45) = 1.94, p = 0.058). Walking speed of the participants with iSCI was significantly lower compared to healthy controls walking in the self-paced (SP) condition (t(41.7) = − 7.35, p < 0.001), but not significantly different from their fixed-speed 50% (FS50) condition (t(32.0) = 1.61, p = 0.116).

**Table 1:** Characteristics of participants with incomplete spinal cord injury (iSCI) and healthy controls (HC) (mean ± SD or median [range])

	iSCI	HC	p
N	28	19	
Sex (M/F)	18/10	9/10	0.250
Age (year)	58 ± 13	60 ± 9	0.611
Weight (kg)	85 ± 13	75 ± 13	0.010
Height (cm)	177 ± 7.9	172 ± 7.0	0.034
BMI	27.2 ± 3.7	25.1 ± 3.3	0.058
Walking speed (m/s)			
Self-paced	0.85 ± 0.37	1.45 ± 0.18	0.000
Fixed-speed 50%		0.74 ± 0.09	0.116*
iSCI characteristics			
AIS (C/D)	2/26		
Level of injury	Thoracic 5 [Cervical 1–Lumbar 4]		
Post-injury (months)	23 [6 212]		
Cause (traumatic/non-traumatic)	3/25		
FAC (3/4/5)	1/5/22		

BMI: body mass index; AIS: American spinal injury association impairment scale; FAC: functional ambulation categories

^*^Indicates the comparison between healthy controls walking in the fixed-speed 50% condition and people with iSCI walking in the self-paced condition

### Foot placement deviation

**Fig. 1:**
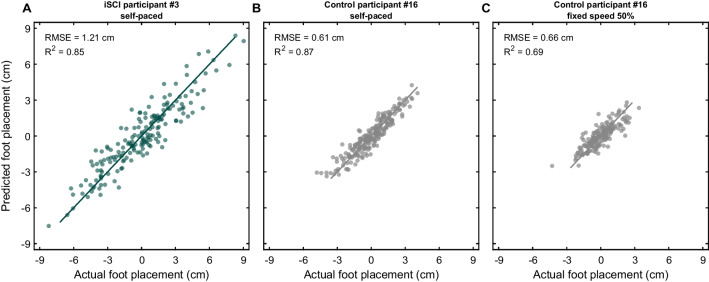
Actual mediolateral (ML) foot placement versus predicted ML foot placement of a person with incomplete spinal cord injury (iSCI) walking in the self-paced condition (**A**) and of a healthy control walking in the self-paced condition (**B**) and the fixed-speed 50% condition (**C**). RMSE indicates the root mean square error between the actual and predicted foot placements, referred to as foot placement deviation. R^2^ indicates the coefficient of determination of the fit, referred to as the foot placement adherence

**Fig. 2:**
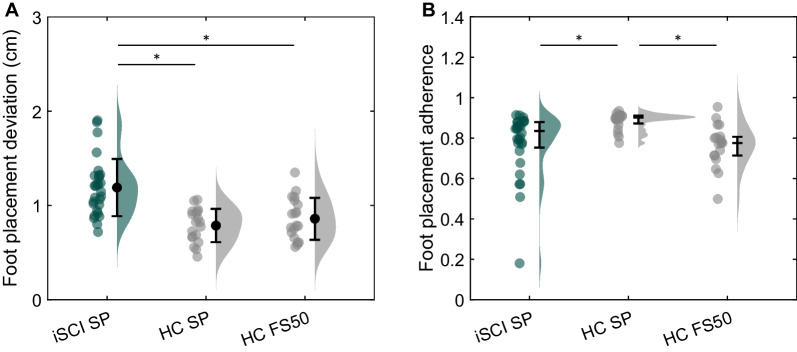
Raincloud plots of foot placement deviation (RMSE) (**A**) and foot placement adherence (R^2^) (**B**) of people with incomplete spinal cord injury (iSCI) and healthy controls (HC) walking in the self-paced (SP) or fixed-speed 50% (FS50) condition. Dots represent the individual datapoints and bars the mean ± standard deviation (A) or median and 25th and 75th percentile (B). *p < 0.017

The actual ML foot placements and predicted ML foot placements of a representative participant from both groups are shown in Fig. 1. At group level, foot placement deviation of people with iSCI walking in the self-paced condition was higher compared to healthy controls independent of walking speed (SP: t(45) = 5.21, p < 0.001; FS50: t(45) = 4.06, p < 0.001; Fig. 2A, Table 2). Participants with iSCI exhibited 0.40 cm (51%) and 0.33 cm (38%) higher foot placement deviation compared to healthy controls walking in the self-paced and the FS50 condition, respectively. No significant difference in foot placement deviation was found between healthy controls walking in the self-paced and the FS50 condition (z = 2.01, p = 0.044).

**Table 2:** Foot placement deviation (RMSE), foot placement adherence (R^2^), standard deviation (SD) of the actual foot placement, and step width of people with incomplete spinal cord injury (iSCI) and healthy controls (HC) walking in the self-paced (SP) or fixed-speed 50% (FS50) condition (mean ± SD or median [range])

	iSCI SP	HC SP	HC FS50
Foot placement deviation (cm)	1.19 ± 0.30 ^*, †^	0.79 ± 0.18	0.86 ± 0.22
Foot placement adherence	0.85 [0.18–0.91] ^*^	0.90 [0.78–0.94] ^‡^	0.78 [0.50–0.95]
SD actual foot placement (cm)	2.78 ± 0.88 ^†^	2.37 ± 0.51 ^‡^	1.90 ± 0.62
Step width (cm)	18.17 ± 5.65 ^*, †^	12.59 ± 2.65	11.44 ± 2.73

Significant differences (p < 0.017) between iSCI SP and HC SP (*), iSCI SP and HC FS50 (^†^), and HC SP and HC FS50 (^‡^)

### Foot placement adherence

All participants except one iSCI participant showed substantial foot placement adherence (R^2^ ≥ 0.26). At group level, foot placement adherence of people with iSCI was lower compared to healthy controls walking in the self-paced condition (z = 3.62, p < 0.001; Fig. 2B, Table 2), but there was no longer a significant difference when people with iSCI were compared to controls in the FS50 condition (z = − 1.19, p = 0.233). In addition, foot placement adherence was lower in healthy controls walking in the FS50 condition compared to walking in the self-paced condition (z = − 3.67, p < 0.001).

### Foot placement variability

**Fig. 3:**
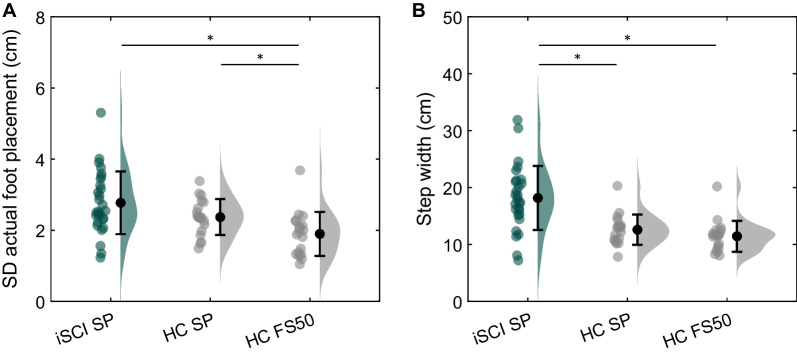
Raincloud plots of the standard deviation (SD) of the actual foot placement (**A**) and step width (**B**) of people with incomplete spinal cord injury (iSCI) and healthy controls (HC) walking in the self-paced (SP) or fixed-speed 50% (FS50) condition. Dots represent the individual datapoints and bars the mean ± standard deviation. *p < 0.017

No significant difference in the SD of actual foot placement was found between people with iSCI and healthy controls walking in the self-paced condition (t(44.0) = 1.99, p = 0.053; Fig. 3A, Table 2). When compared to healthy controls walking in the FS50 condition, participants with iSCI exhibited 0.88 cm (46%) higher SD of actual foot placement (t(45) = 3.74, p < 0.001). Moreover, healthy controls walking in the self-paced condition exhibited 0.47 cm (25%) higher SD of actual foot placement compared to walking in the FS50 condition (t(18) = 4.18, p < 0.001).

### Step width

Step width of people with iSCI was higher compared to healthy controls independent of walking speed (SP: t(40.9) = 5.45, p < 0.001; FS50: t(41.4) = 5.44, p < 0.001; Fig. 3B, Table 2). Participants with iSCI walked with 5.59 cm (44%) and 6.73 cm (59%) wider steps compared to healthy controls walking in the self-paced and the FS50 condition, respectively. No significant difference in step width was found between healthy controls walking in the self-paced condition and the FS50 condition (t(18) = 2.33, p = 0.032).

## Discussion

In the current study, we found that—independent of walking speed—the accuracy of the ML foot placement strategy during walking in people with iSCI was reduced compared to healthy controls.

### Foot placement deviation

Congruent with our hypothesis, people with iSCI showed significantly higher foot placement deviation compared to healthy controls, indicating an impaired ML foot placement strategy. This finding is in line with the results of Cornwell et al. [6] and Day et al. [4], who found indications of a weaker coordination between COM state and lateral foot placement in individuals with iSCI. An impaired ML foot placement strategy in people with iSCI could be explained by two important underlying mechanisms. The first mechanism is the impaired proprioceptive information from body structures below the lesion level [1] that could impede the estimation of the COM state and the spatial location of the feet. Indeed, changes in ML foot placement during walking have been observed in healthy subjects when proprioceptive information from muscle spindles was manipulated through muscle vibration [35]. The second mechanism is the decreased muscle coordination in people with iSCI [1], which affects the ability to control the swing leg and therefore limits the coordination of foot placement. In line with this notion, previous research observed a smaller magnitude of soleus activation in the swing leg after slip perturbations in people with iSCI compared to healthy controls [7], implying impaired muscle control in balance-challenging conditions. Moreover, decreased coordination of foot placement has been observed in people with stroke performing a hip abduction tracking task [36], suggesting that reduced control of the swing leg may limit coordination between COM movement and foot placement. With the current study, we cannot determine to what extent impaired proprioceptive information and/or decreased muscle coordination underly the impaired foot placement strategy in people with iSCI. Therefore, future research should focus on disentangling the role of both mechanisms on the foot placement strategy, which can help design and optimize interventions for people with iSCI. Nevertheless, current interventions could focus on provoking more lateral COM excursion and velocity to specifically train the coordination between COM kinematics and ML foot placement. Examples of such interventions are perturbation-based balance training [37] or walking adaptability training [38].

### Foot placement adherence and variability

All participants except one participant with iSCI showed substantial adherence to the foot placement strategy, indicating that both people with iSCI and healthy controls use the foot placement strategy during walking. People with iSCI had significantly lower foot placement adherence compared to healthy controls walking at a self-paced speed. However, when corrected for walking speed, the significant group difference in foot placement adherence disappeared. It should be acknowledged that foot placement adherence is influenced by the within-subject SD of actual foot placement, i.e., a larger SD of actual foot placement results in larger foot placement adherence. In line with previous research [4], participants with iSCI walking at a self-paced speed showed a significantly larger SD of actual foot placement compared to healthy controls walking at 50% of their preferred speed (see Figs. 1 and 3A). As a result, a valid comparison of foot placement adherence between both groups is hard to make.

### Step width

Participants with iSCI had a larger step width compared to healthy controls. Increased step width has previously been linked to instability during walking [32, 33]. Healthy subjects increased step width when perturbed in the ML direction [39] or while walking on a destabilizing surface [40]. In contrast, individuals with iSCI decreased step width when walking stability was increased by external lateral stabilization [9]. Moreover, healthy subjects decreased modulation of foot placement based on the COM state in response to an increased (imposed) step width [34]. These results suggest that wider steps increase postural stability and therefore reduce the demand for accurate foot placement modulation. Therefore, it is likely that people with iSCI increased their step width to improve postural stability, thereby compensating for a decreased foot placement strategy.

### Effect of walking speed

The walking speed of the participants with iSCI was significantly lower compared to healthy controls walking in the self-paced condition. Therefore, an effect of walking speed on the foot placement strategy should be considered. Healthy controls showed similar foot placement deviation while walking at 50% of their preferred walking speed compared to walking at a self-paced (preferred) speed, suggesting no effect of walking speed on the ML foot placement strategy. In the literature, conflicting results regarding the effect of walking speed on the ML foot placement strategy have been reported. Wang and Srinivasan [19] found no effect of walking speed on the prediction of ML foot placement based on the upper body state. Likewise, Stimpson et al. [41] observed that speed-dependent differences in the ML foot placement strategy largely disappeared at the end of a step. In contrast, Cornwell et al. [6] and van Leeuwen et al. [30] found a stronger correlation between COM state and ML foot placement at fast walking speeds. Of note, all studies assessed foot placement adherence (R^2^) to evaluate the foot placement strategy. As mentioned before, foot placement adherence is influenced by the within-subject SD of actual foot placement. Because this latter parameter increases at faster walking speeds [6, 41, 42], the effect of walking speed on foot placement adherence is hard to extrapolate.

### Limitations

Participants with iSCI were included if they were able to walk in a self-paced mode on the GRAIL without using the handrails. This resulted in a group of individuals who were mild to moderately affected. Therefore, the results cannot be generalized to all individuals with iSCI. As a higher impairment level in iSCI potentially affects their sensorimotor control more, it can be expected that the ML foot placement strategy is more severely impaired in individuals with a higher impairment level. Further research is necessary to evaluate the relation between the ML foot placement strategy and the level of impairment in people with iSCI.

Healthy controls walking in the FS50 condition exhibited 0.47 cm lower SD of actual foot placement compared to walking in the self-paced condition. This decrease in SD of actual foot placement when walking in the FS50 condition could be the result of a difference in treadmill mode (i.e., fixed speed versus self-paced). Yet, Sloot et al. [21] showed that step width variability increased with only 1 mm when walking in a fixed-speed mode compared to a self-paced mode. Therefore, we anticipate that the treadmill mode itself had only little effect on the SD of actual foot placement.

Finally, the healthy controls in this study were not sex- or age-matched to the participants with iSCI. Nevertheless, we aimed to include healthy controls in a similar age category as the majority of people with iSCI (age ≥ 45 years) [43]. Furthermore, we found no significant difference in age and sex between both groups.

## Conclusion

This study found a higher foot placement deviation in people with iSCI compared to healthy controls independent of walking speed, indicative of an impaired ML foot placement strategy. Moreover, our results suggested that people with iSCI tended to compensate for this decreased foot placement strategy by increasing their step width. Future research should focus on improving the foot placement strategy by targeted balance training.

## Data Availability

The data used and analyzed during the current study are available from the corresponding author on reasonable request.

## Abbreviations

AIS: American spinal injury association impairment scale
ASIS: Anterior superior iliac spine
BMI: Body mass index
BOS: Base of support
COM: Center of mass
FAC: Functional ambulation categories
FP: Foot placement
FS50: Fixed-speed 50%
GRAIL: Gait real-time analysis interactive lab
HC: Healthy controls
iSCI: Incomplete spinal cord injury
ML: Mediolateral
RMSE: Root mean square error
PSIS: Posterior superior iliac spine
SD: Standard deviation
SP: Self-paced
2MWT: Two-minute walk test
